# Processing Information about Support Exchanges in Close Relationships: The Role of a Knowledge Structure

**DOI:** 10.3389/fpsyg.2016.00259

**Published:** 2016-02-29

**Authors:** Bulent Turan

**Affiliations:** Department of Psychology, University of Alabama at BirminghamBirmingham, AL, USA

**Keywords:** trust, knowledge, script, responsiveness, social support, close relationships

## Abstract

People develop knowledge of interpersonal interaction patterns (e.g., prototypes and schemas), which shape how they process incoming information. One such knowledge structure based on attachment theory was examined: the secure base script (the prototypic sequence of events when an attachment figure comforts a close relationship partner in distress). In two studies (*N* = 53 and *N* = 119), participants were shown animated film clips in which geometric figures depicted the secure base script and asked to describe the animations. Both studies found that many people readily recognize the secure-base script from these minimal cues quite well, suggesting that this script is not only available in the context of specific relationships (i.e., a relationship-specific knowledge): The generalized (abstract) structure of the script is also readily accessible, which would make it possible to apply it to any relationship (including new relationships). Regression analyses suggested that participants who recognized the script were more likely to (a) include more animation elements when describing the animations, (b) see a common theme in different animations, (c) create better organized stories, and (d) later recall more details of the animations. These findings suggest that access to this knowledge structure helps a person organize and remember relevant incoming information. Furthermore, in both Study 1 and Study 2, individual differences in the ready recognition of the script were associated with individual differences in having access to another related knowledge: indicators suggesting that a potential relationship partner can be trusted to be supportive and responsive at times of stress. Results of Study 2 also suggest that recognizing the script is associated with those items of an attachment measure that concern giving and receiving support. Thus, these knowledge structures may shape how people process support-relevant information in their everyday lives, potentially affecting relationship outcomes and mental and physical health.

## Introduction

A basic tenet of the social-cognitive model is that people develop detailed knowledge of interpersonal interaction patterns, and these knowledge structures shape how they process new information (Baldwin, 1992; Fehr, 2005). These knowledge structures (e.g., schemas, prototypes, scripts) filter incoming information and affect a person's cognitive, behavioral, and affective responses. That is, people do not directly respond to situations themselves; they first process incoming information using existing knowledge structures relevant to the present situation. For example, in one study, participants were shown a video of two people in a living room discussing issues related to crime (Zadny and Gerard, 1974). Participants were told that the video portrayed one of three situations: burglary, searching for drugs, or waiting for a friend in the friend's apartment. Participants formed schemas depending on which of these three interpretations were given to them and the schema affected the way they processed the information in the video. For example, participants who had been led to a burglary schema later recalled more theft-related objects and theft-related dialogs in the video.

Abelson (1981), Schank (1999), Schank and Abelson (2013) developed the notion of a *script* as one type of knowledge structure that helps people to mentally represent *prototypic event sequences*. The restaurant script, for example, lists the order of events when one eats at a restaurant. It has been suggested that the notion of scripts can be applied to interpersonal relationships to examine scripts representing interpersonal interaction patterns (Baldwin, 1992). Waters and Waters (2006) applied the notion of a script to attachment theory and close relationships, and formulated the concept of a secure base script, which is presumed to underlie a secure attachment relationship. This script concerns the prototypic sequence of events in an interaction between two people, in which an attachment figure helps allay the distress of a person. The script contains the following components (or sequence of events): (a) Two partners are engaged in an activity (either together or separately). (b) One person shows signs of distress and (c) looks to the partner for help. (d) The partner notices the person's distress, (e) helps solve the problem, and (f) comforts the person, so that (g) the once-distressed person can now return to normal activities.

In the two studies presented in this article, each person's tendency to recognize the secure base script from minimal cues and to use this script to interpret incoming information was assessed. An ability to recognize the secure base script should shape how people process support-relevant information in their everyday lives, potentially affecting relationship outcomes. Waters and Waters (2006), as well as Mikulincer et al. (2009), have also developed assessment techniques based on the concept of the secure base script, and provided evidence that an ability to provide good exemplars of the secure base script in narrative form is associated with a secure attachment pattern. Both of these approaches differ from the one used in this study in an important way: They involve giving participants strong cues about the script and assessing individual differences in *generating rich and elaborated* secure base narratives, which should be associated with the person's ability to apply the script in existing relationships to establish secure attachments. In contrast, the method used in this article involved creating animated film clips, in which geometric figures depicted the secure base script, with the aim of assessing the degree to which participants recognize the script from minimal cues (and not necessarily to provide a rich narrative), and examining the effect of script-use on the encoding of incoming information.

The term *knowledge structure* implies an internally organized set of elements that affect memory and information processing. Therefore, adapting experimental methods from cognitive psychology, this article illustrates how the secure base script may affect information processing. I also hypothesized that there are individual differences in the degree to which people recognize and use the secure base script, and examined the association between having access to this script and having access to a second knowledge structure closely related to support exchanges in close relationships—indicators of a partner who is apt to be there when needed. Turan and Horowitz (2007, 2010), Turan (2011) developed a method to assess individual differences in having access to this knowledge structure (indicator knowledge) and provided evidence for its validity as an individual difference measure.

Both knowledge structures help a person evaluate the likelihood that a partner will be there when needed (i.e., trusting a partner to be supportive; Rempel et al., 1985). Indicator-knowledge concerns behavior patterns and traits that a responsive person exhibits; script-knowledge articulates the prototypic sequence of events that constitute the situations in which those behaviors get expressed. In other words, recognizing the script enables a person to evaluate a partner's behavior patterns and traits in context—i.e., a person-by-situation interaction (Zuroff, 1982; Idson and Mischel, 2001). As Holmes (2002) has noted, “One can only identify the *person* as a figure against the ground of the situation” (p. 8). Because these two types of knowledge jointly contribute to judgments—and because they are presumably derived from similar past experiences—it is expected that people with better indicator-knowledge are also more likely to recognize the secure base script. Because the two knowledge structures are, theoretically, part of a higher-order construct—social competence in understanding support processes in close relationships—there should be an association between the degree to which people (a) recognize the secure base script and (b) possess indicator-knowledge. In addition to replicating and extending the findings of Study 1, Study 2 examined the association between recognizing the secure base script in geometric figures and attachment styles and generalized expectations for support from close relationship partners. The ability to recognize the secure base script may help a person to recognize when a partner is apt to provide quality support and to benefit more from support exchanges.

To summarize, the hypotheses of the present studies were:

1. Many people readily recognize the secure-base script from minimal cues, suggesting that this script is not only available in the context of specific relationships (i.e., a relationship-specific knowledge): The generalized (abstract) structure of the script is also readily accessible, which would make it possible to apply it to any relationship (including new relationships).
2. Recognizing the script affects information processing. Therefore, participants who recognize the script are more likely to (a) see a common theme in different animations, (b) include more elements when describing the animations, (c) create better organized descriptions of the animations, and (d) later recall more details of the animations.
3. Individual differences in the ready recognition of the script is associated with individual differences in having access to the knowledge of indicators suggesting that a potential relationship partner can be trusted to be supportive and responsive at times of stress.

## Study 1: recognizing the secure base script in animated film clips

Baldwin (1992) proposed that the availability and accessibility of cognitions concerning close relationships shape how people respond to incoming interpersonal information. An interesting theoretical question about the availability/accessibility of the secure base script is the following: Do people readily recognize the secure base script only in their relationships with certain other people (a *relationship-specific* knowledge)? Or, can people recognize the *generalized* (abstract) structure of the script that applies to *any* relationship? It may be argued that in order to use the knowledge of the script in new situations with new relationship partners, among other things, the person must be able to recognize the *abstract* (content free) structure of the secure base script—the *common, invariant sequence of events* across different instances (realizations) of the script (cf., Gentner and Markman, 1997; Schank, 1999).

Because of the importance of the secure base script in developing close relationships, I hypothesized that many people are able to recognize the abstract script structure quite well even when it is completely stripped of concrete content. Therefore, I developed a technique to assess the degree to which participants recognize the abstract structure of the secure base script from minimal cues—when it is stripped down to its temporal/causal foundation. The best approach to achieve this goal would be to use stimuli that do not involve any human content. To this end, three animated film clips were created. These animations portray simple geometric figures, such as circles and triangles. The geometric figures enact the sequence of events that suggest the secure base script, and participants are asked to describe what they think is happening in the animations. This method was suggested by the classic animation studies by Heider and Simmel (1944) and Michotte (1963).

A coding scheme was developed to score the extent to which participants' descriptions of the animations incorporated the secure base script. These scores were used to assess the degree to which each participant recognizes and uses the abstract structure of the script. Note that using the secure-base script in interpreting incoming information is distinct from attachment security, which reflects an expectation that attachment figures will be available when needed and will provide support in an effective way. Participants either used the secure-base script to interpret the animations or they used other scripts such as dancing or enjoying a party; no participant described the animations in terms of an insecure attachment relationship. Using the secure-base script is also distinct from *generating rich and elaborated* attachment/support narratives in response to stimuli that clearly depict a distressing situation (Waters and Waters, 2006; Mikulincer et al., 2009). The animation procedure simply assesses whether or not the participant readily applies the script of giving/receiving support to a distressed person.

An important property of a script is that it affects how people process relevant information. For example, research has shown that people remember previously presented material better if they can rely on a script to mentally organize the material (Bower and Clark, 1969; Graesser et al., 1980). If a sophisticated script exists to represent the sequence of events concerning support exchanges, then this script should help a person to organize perceptions of relevant events and affect how the person perceives, interprets, and remembers relevant stimuli—provided that the script is activated.

Therefore, I expected that participants who are more likely to use the secure base script to understand the animations are more likely to recall the details of the animations, because the organizing property of the script should help them find meaning in otherwise ambiguous movements in the animations. Similarly, participants who recognize the script should be able to describe the animations in a more coherent way. If the animations are indeed best understood in terms of the secure base script, descriptions incorporating that script would be better organized in terms of causal connections.

A person who recognizes the abstract structure of the secure base script should also be able to perceive the sequence of events that is common to all three animations. Therefore, participants were also asked to describe what they thought was the *common theme* in all three animations. The degree to which a participant articulated the secure base script as the common theme constituted another measure of that participant's ready recognition of the script. I hypothesized that these two measures of script-recognition would be associated with each other. I also tested the hypothesis that recognizing the script is related to participants' indicator-knowledge.

### Methods

#### Participants

Participants were undergraduate students in introductory psychology courses (28 men, 25 women), who participated to fulfill the requirement for research participation. Participants signed up for an available time slot using an online system. Participants' mean age was 20.9 (*SD* = 5.7). Twenty-four were Caucasian, 12 Asian-American, 5 Hispanic, and 9 African-American.

#### Animations (film clips)

Three animations depicted simple geometric figures interacting with each other. Each animation, lasting exactly 30 s, portrays a clear example of the secure base script. Pilot testing suggested that some animations could be described using scripts other than the secure base script. That is, some participants described those animations using other plausible scripts. Therefore, those animations were modified so that the alternative description was no longer plausible. My goal was to create animations that are best interpreted as examples of the secure base script. I assumed that participants who readily recognize the abstract structure of the secure base script would favor that interpretation over less plausible interpretations.

**Figure 1:**
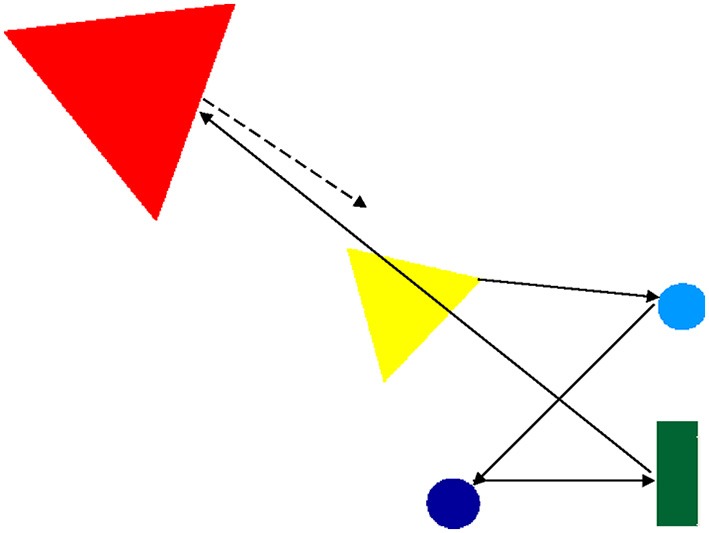
**Snapshot of a sample secure base animation**. The arrows show the yellow triangle's movements up to the point where it meets with the red triangle (the attachment figure) to be comforted. Then, the yellow triangle will go back to its original position (lower right corner where two circles and a rectangle are located), but these movements are not shown by the arrows (to keep the illustration simple).

In one animation, for example, a yellow triangle approaches other geometric forms—two circles and a rectangle (representing the play/exploration step of the script, see Figure 1). After it touches the rectangle, the triangle starts to vibrate rapidly (i.e., a sign of distress). Then it approaches a larger (red) triangle, makes contact with it, and together they turn right and left (i.e., the small triangle is comforted by the support figure). Then, the small triangle goes back to the original geometric forms and touches them one by one (a return to exploration/play). When it touches the rectangle this time, it does not vibrate; instead its tip stays in contact with the rectangle while the triangle turns about 45°.

#### Measures

##### The knowledge of indicators (KNOWI) task (Turan and Horowitz, 2007, 2010; Turan and Vicary, 2010; Turan, 2011)

The KNOWI Task was developed to assess individual differences in knowledge of the good indicators that a partner is apt to be there when needed. The scale contains 11 good indicators, 11 poor indicators, and 19 filler items. An example of a good indicator is “notices changes in my mood and asks if anything is wrong.” An example of a poor indicator is “does not ignore others on the street.” An example of a filler item is “is friendly to everyone.” Participants rate the degree to which each indicator would “increase your confidence that a potential (hypothetical) partner will “be there” for you” (using a scale 1–8). The task was constructed to be analogous to a signal detection task, where participants have to discriminate signal (good indicators) from noise (poor indicators). Each participant's ratings are averaged separately for the good (G) and poor (P) indicators. A participant's accuracy index is defined as the *difference* between that participant's two means, denoted G – P. Turan and Horowitz (2007, 2010), Turan and Vicary (2010), Turan (2011) presented data from laboratory and questionnaire studies that support the validity of the accuracy index of the KNOWI as a measure of knowledge of good indicators of a partner who is apt to be there when needed.

A participant's criterion bias (response readiness or KNOWI-readiness) is defined as the *sum* of that participant's two means (G + P). It reflects the participant's motivation for support (Turan and Horowitz, 2010; Turan and Vicary, 2010). When examining the effect of KNOWI-accuracy, KNOWI-readiness is always entered as a control variable. In this study, the KNOWI-accuracy and KNOWI-readiness scores were used the same way as in all previous studies using the KNOWI.

### Procedure

This study received ethical approval from Stanford University Institutional Review Board and all procedures were carried out with the adequate understanding and written consent of the participants. Participants were tested individually. Using a computer, the experimenter presented each animation twice. After the second viewing, participants were allowed 120 s to type their description of the animation. The participants first practiced the entire procedure with a practice clip. Then they were given the three secure base animations in a fixed order. After they finished typing their description of the last animation, the experimenter asked them if they thought that there was a common theme to all three animations. The participants were allowed 120 s to type what they thought the common theme/story was.

Then participants performed two interpolated activities for 5 min. The first was a memory task unrelated to the present experiment; the second was a demographic questionnaire. Next, the experimenter instructed the participant: “Please write what you remember from the clips. Describe *literally* what happened, e.g., the blue circle moved toward the red rectangle.” The participants received a printout of the first scene of each animation, and they had 90 s to type their recall responses for each animation. Then the participants performed another interpolated task unrelated to the present study, and finally they completed the KNOWI Task.

#### Coding the responses

##### Coding the descriptions for the secure base script

A coding scheme was developed to score participants' descriptions in terms of the degree to which they followed the secure base script. The coding scheme explains how each specific animation portrays the script. Instructions are given to coders to specify under what conditions each of the scale points (1–7) should be assigned to a description. Using this coding scheme three coders independently rated descriptions for the three animations as well as responses to the common theme question provided by the participants. The ratings reflect the degree to which a description (or a common theme response) incorporates the secure base script. To earn a high rating, a response has to incorporate all elements of the script in the correct order. Interrater reliability was good; intraclass correlations for the three raters' ratings were 0.97, 0.93, 0.95, and 0.96, respectively, for clips 1, 2, 3, and the common theme. The ratings for the description of each animation, averaged across raters, were then averaged across the three animations to provide an overall measure of the degree to which each participant' recognized the secure base script (Cronbach's alpha for the three animation descriptions was satisfactory; α = 0.64). In addition, the ratings for the common theme were also averaged across the raters to provide a second measure of the same construct.

##### Coding the recall

First, two undergraduate research assistants independently watched each animation and identified each separate “action unit” that occurred in that animation. An example of an action unit was, “the yellow triangle moved toward the turquoise circle.” Then they compared their lists of action units and finalized the list of action units for each animation.

Two additional coders (both graduate students, who were not familiar with the concept of the secure base script or the hypotheses of the study) then independently read each participant's recall responses and, for each animation, counted the number of listed action units that the participant was able to recall correctly. Inter-rater reliability was good; intraclass correlations for the two raters were 0.85, 0.84, and 0.93 for animations 1, 2, and 3, respectively. These scores were averaged across raters, and then averaged across the three animations to provide a measure of each participant's ability to recall literal details contained in the original animations (Cronbach's alpha for the recall of the three animations was satisfactory; α = 0.77).

##### Coding for temporal continuity, causal connections, and organization of the original descriptions

If the animations are best conceptualized in terms of the secure base script, descriptions incorporating that script should be better organized in terms of temporal continuity and causal connections. To test this hypothesis, two additional undergraduate research assistants (both unfamiliar with the secure base script and the hypotheses of the study) coded the quality of the organization of the descriptions, using a coding scheme adapted from the one Waters and Hou (1987) developed. First, the coders rated the temporal continuity of the descriptions using a 3-point scale. Descriptions received high scores if there was a strong temporal order of the sentences so that if the order of the sentences were to be switched, the meaning of the description would change substantially. Second, they counted the number of causal connections a description included. An example of a causal connection was: “The kid lost his ball, so he got upset.” Finally, the coders rated (using a 5-point scale) the overall organization of the descriptions by considering how many temporal and causal/goal-directed sentences the participant used. These coders were not familiar with the secure base script and did not examine the secure base script ordering of events. Rather, they coded for *any* temporal order or causal connections.

Intraclass correlations were computed to examine inter-rater reliability on these ratings. Intraclass correlations ranged from 0.74 to 0.88 for the organization scores, from 0.78 to 0.86 for the causal connections, and from −0.06 to 0.82 for the temporal connections. These intraclass correlations suggest that coders agreed well in scoring for organization and causal connections, but not in scoring temporal connections. Further analyses suggested that the reason for low inter-rater reliability in judging the temporal connections was due to lack of variability in these scores; almost all participants were able to describe the animations using a strong temporal order. Therefore, only results related to organization and causal connections will be discussed.

### Results and discussion

#### Description of animations (secure base coding)

Participants were quite successful in recognizing the secure base script in the animations. A sample response that received high scores from the raters was:

The triangle was exploring an area, and it felt safe when coming into contact with the two circles. However, when it came across the green rectangle, it became frightened and shook. The square noticed this reaction and moved toward the triangle. The square rocked the triangle to comfort it. The triangle felt safe to go explore the rectangle, then.

Surprisingly, however, not everyone seems to recognize the secure base script readily. Some participants could not see any meaningful story depicted by the animations. Still other participants could tell a story, but that story did not incorporate the secure base script at all.

The mean scores for the description of the three animations (reflecting the degree to which the descriptions followed the secure base script) showed considerable variability, ranging from 1.0 to 6.2 (*M* = 3.4, *SD* = 1.6). Similarly, the common theme scores ranged from 1.0 to 7.0 (*M* = 3.3, *SD* = 2.0). As expected, these two measures correlated very highly with each other, *r* = 0.79, *p* < 0.001, suggesting that they assess the same construct (recognizing the secure base script).

#### Recall of animations

On average, participants recalled about half of the action units (*M* = 0.55, *SD* = 0.12, for the proportion of action units correctly recalled). To examine the hypothesis that the two measures of participants' ready recognition of the secure base script (the mean of the three clip scores and the common theme score) predict the proportion of the action units that they recalled, two separate regression analyses were conducted. These analyses revealed a significant association between the mean of the three clip scores and recall (β = 0.56, *t* = 4.77) and a significant association between common theme scores and recall (β = 0.57, *t* = 4.91; both *p*s < 0.001). These results suggest that participants who more readily recognized the secure base script were also better able to recall literal elements of the animated film clip. Thus, the organizing property of the script seems to help a person retrieve details of an otherwise ambiguous stimulus.

It is possible that differences in recalling the animations were present even immediately after viewing them and that these differences in recall affected script-recognition. This alternative explanation suggests that the causal relationship between recognizing the script and recall is in the opposite direction. However, it should be noted that the participants watched each animation *twice*, and immediately after the second viewing of a particular animation they typed what they thought was happening in that animation, which makes it less likely that there were big differences in memory at that point.

#### Causal connections and organization

To test the hypothesis that the two measures of participants' ready recognition of the secure base script (the mean of the three clip scores and the common theme score) are associated with the overall organization of their descriptions, I conducted regression analyses predicting the scores on the overall organization of the descriptions averaged across the three animations. The mean scores on recognizing the secure base script across the three animations (secure base scriptedness scores of the descriptions) was a significant predictor (β = 0.63, *t* = 5.75, *p* < 0.001). In a separate regression analysis, the common theme score was also a significant predictor (β = 0.51, *t* = 4.20, *p* < 0.001). That is, participants who recognized the secure base script in the animations provided descriptions that were better organized. A second set of regression analyses were conducted to predict the mean causal connection scores across the three animations. The mean scores on recognizing the secure base script across the three animations (secure base scriptedness scores of the descriptions) was a significant predictor (β = 0.65, *t* = 6.13, *p* < 0.001). In a separate regression analysis, the common theme score was also a significant predictor (β = 0.49, *t* = 3.99, *p* < 0.001).

Thus, results of Study 1 suggest that some people readily recognize and use a well delineated knowledge structure, which can be summarized as the secure base script. Many participants could detect the script simply from the sequence of events depicted by geometric figures, which do not include any human content. Participants could also recognize the higher-order abstraction from structural similarities among the three animations. The recognition of this abstract script structure, in turn, seems to shape how they organize their perceptions of the stimuli in the animations, affecting their memory for these stimuli and the organization of their descriptions of the animations. Thus, it seems that ready recognition of the secure-base script is not limited to the context of relationships with specific close relationship partners (i.e., a relationship-specific knowledge): People can also recognize the generalized (abstract) structure of the script that can apply to any relationship (including new relationships). These results suggest the possibility that using the secure base script may shape how people process support relevant information in their everyday lives—how they interpret and remember supportive efforts of relationship partners and how they make global judgments about partners. This may account for the recent findings on the associations between individual differences in these knowledge structures and relationship outcomes (Turan and Vicary, 2010; Turan et al., 2011).

#### Script recognition and indicator knowledge

As hypothesized, in separate regression analyses both measures of ready recognition of the secure base script were significantly associated with the KNOWI-accuracy scores (controlling for KNOWI-readiness as was done in other studies using the KNOWI); β = 0.33, *t* = 2.69, *p* = 0.01, and β = 0.33, *t* = 2.65, *p* = 0.01, respectively, for the mean of the three clip scores and the common theme score. This result supports the hypothesis that people with greater knowledge about indicators of a partner who is apt to be there when needed (high scores on KNOWI-accuracy) are more likely to recognize the secure base script, suggesting that these two knowledge structures are related, perhaps because they are part of a higher-order cognitive structure related to making judgments about trusting a partner to be there when needed. It is possible, however, that general intelligence or a general ability to impose human qualities onto geometric forms (even when the content of the animation is unrelated to the secure base script) may have caused or inflated the associations with ready recognition of the secure base script.

## Study 2: Recognizing the secure base script, recognizing other scripts, and indicator knowledge

Study 2 tested the generality and specificity of the findings of Study 1 in two ways. First, in Study 1 only three secure base animations had been used; there were no control animations depicting scripts other than the secure base script. It is possible that the animation task simply assesses a general ability to impose human qualities onto geometric forms (even when the content of the animation is unrelated to the secure base script).Second, the KNOWI Task in Study 1 had been administered in the laboratory immediately after the animation task, possibly augmenting the effect. Therefore, it was important to demonstrate that the results did not depend on the simultaneous administration of the two tasks.

To address these issues, the procedure was modified in Study 2. Three “control” animations were interspersed among the experimental (secure base) animations. These animations served as control stimuli depicting scripts other than the secure base script—e.g., a race between people. Responses to these control stimuli were used to assess each participant's ability to create stories describing how geometric figures might interact in human-like ways. It was then possible to determine whether the participants' performance on the secure base animations was related to their indicator-knowledge of supportiveness (scores on the KNOWI Task), controlling for their ability to describe animations in human-like ways. In addition, the KNOWI was administered several weeks before the laboratory session.

Study 2 also examined associations between individual differences in these two knowledge structures and other interpersonal constructs, such as attachment styles, generalized expectations for support in relationships, and motivation for support in relationships. Most items in our attachment measure do not concern giving or receiving support in times of need (e.g., “I find it relatively easy to get close to my partner”). Therefore, we also examined associations with the items of the attachment measure that concern giving or receiving support in times of need (i.e., being there, which is the focus of the present article). A sample item that does concern seeking support is: “I turn to my partner for many things, including comfort and reassurance.” Shaver et al. (2000) findings also point to the importance of being there for a partner in times of need as a primary component of attachment relationships. These researchers found that self-report items that reflect relying on a partner's support at times of need are most highly associated with an interview measure of attachment security. Similarly, Collins and Read (1990) showed that seeking the support of others at times of need is an important (and distinct) component of attachment security.

One could also argue that attachment orientations related to support exchanges depend on knowledge about support processes and motivation and expectations regarding support processes. Therefore, a supplemental regression analysis was performed predicting the attachment items concerning being there using the following independent variables: (a) desire (motivation) for support, (b) generalized expectations for support, (c) knowledge of indicators of supportiveness, and (d) knowledge of the secure base script. The goal with these exploratory analyses was to explore the possibility that attachment concerns related to support can be broken down into different, theoretically meaningful parts.

### Methods

#### Participants

The participants were 119 undergraduate students in introductory psychology courses (who did not take part in Study 1) and participated to fulfill the requirement for research participation (74 women, 44 men). Their mean age was 19.4 years (*SD* = 1.37). 55 were Caucasian, 26 Asian-American, 15 Hispanic, and 6 African-American.

#### Measures

##### The knowledge of indicators (KNOWI) scale (Turan and Horowitz, 2007, 2010; Turan and Vicary, 2010; Turan, 2011)

Every participant had completed the KNOWI Scale (described in detail under Study 1) as part of a questionnaire packet administered earlier in the term. The accuracy index (KNOWI-accuracy) assesses participants' knowledge of the indicators of supportive people. The response readiness index (KNOWI-readiness) reflects the strength of the participants' motivation for supportive relationships (Turan and Horowitz, 2007, 2010). Both indices were used in the present study.

##### Experiences in close relationships (ECR; Brennan et al., 1998)

The 36-item ECR is widely used to assess the two dimensions of attachment, namely, anxiety (18 items) and avoidance (18 items). An item of the anxiety scale is “I worry about being abandoned,” and an item of the avoidance scale is “I try to avoid getting too close to my partner” (rated using a scale 1–7). A subset of participants (*n* = 61) completed the ECR as part of a questionnaire packet administered early in the term. In the present data, both scales showed good internal consistency; alpha = 0.93 and alpha = 0.89, respectively, for the avoidance and anxiety scales.

##### Generalized expectations for support

This measure was constructed for the present study. Participants were asked to rate their agreement with 16 statements. Half of the statements concerned participants' perceptions of *most relationships*, such as, “In romantic relationships, most people can go to their partner when upset.” The other half of the items asked participants to predict how their *next* partner would behave; a sample item was, “I predict that in my next romantic relationship my partner will not really be there for me when I'm in trouble” (reverse coded). The wording of the items were adapted from existing scales on trust and secure base support (e.g., Rempel et al., 1985; Woodhouse et al., 2009). In the present data, alpha was 0.83.

#### Animations

There were four secure base animations (clips). In addition, three control animations (also with geometric figures, each lasting exactly 30 s). Each control animation depicted the sequence of events making up a different script—one animation was about a footrace, another about the game “duck, duck, goose,” and a third depicted the story of “the hare and the tortoise.” The control animations were constructed in such a way that they were comparable to the secure base animations in their clarity in representing the underlying script. That is, they were comparable in how easy (or difficult) they were for participants to recognize the underlying scripts—many, but not all, participants could recognize the relevant scripts.

### Procedure

This study received ethical approval from Stanford University Institutional Review Board and all procedures were carried out with the adequate understanding and written consent of the participants. Participants were tested individually using procedures similar to those in Study 1. First, they watched a practice animation, then a secure base animation, then a control animation. Subsequent stimulus clips alternated between secure base and control animations. After viewing an animation twice, the participants described what they thought was happening. The instructions were identical to those used in Study 1. The participants had 90 s to type their responses.

#### Coding of the descriptions for secure base scriptedness

Three coders rated descriptions of the secure base animations, using the same coding scheme as in Study 1. Intraclass correlations ranged from 0.92 to 0.97 for the four secure base animations. The three coders' ratings were then averaged for each animation. In the analyses below, each participant's mean score for the four secure base animations was used as a measure of that participant's ready recognition of the secure base script. Similarly, three coders independently rated how well the participants could recognize the different scripts depicted in the control animations (intraclass correlations ranged from 0.94 to 0.98). These ratings were then averaged across the three control animations and across the three coders to obtain a measure of each participant's general ability to recognize scripts in this type of animations. It should be noted that participants almost never used the secure base script in describing the control animations, indicating that script-knowledge is domain specific.

#### Coding for causal connections and organization

Two undergraduate research assistants (neither had participated in secure base scriptedness coding) coded the quality of the organization of the descriptions (adopting the coding scheme developed in Study 1). First, they counted the number of causal connections a description contained. Then, (using a 5-point scale) they rated the overall organization of the descriptions by counting how many causal/goal-directed sentences the participant had used. Inter-rater reliability (intra-class correlations) ranged from 0.80 to 0.86 for the organization scores, and from 0.82 to 0.89 for the causal connections.

#### Coding for the number of action units used in describing the animations

I wanted to determine how much of the actual content of each animation had been used by each participant in describing a secure base animation. Two coders independently counted the number of action units that the participant had used in describing each animation. Inter-rater reliability was good; intraclass correlations for the two raters were 0.97, 0.71, 0.83, and 0.98 for the four secure base animations.

### Results and discussion

#### Association between script scores and Knowi scores

The mean scores for the description of the four secure base animations (reflecting the degree to which the descriptions followed the secure base script) ranged from 1.13 to 7.0 (*M* = 4.01, *SD* = 1.3). The mean scores for the description of the three control animations ranged from 1.25 to 6.92 (*M* = 3.96, *SD* = 1.4). As hypothesized, indicator-knowledge (KNOWI-accuracy, controlling for KNOWI-readiness) was significantly associated with secure base script-recognition; β = 0.23, *t* = 3.25, *p* = 0.002. However, KNOWI-accuracy scores were not significantly associated with scores on the control animations; β = −0.02, *t* = 0.25, *p* = 0.80 (again controlling for KNOWI-readiness). The association between KNOWI-accuracy and scores on the secure base animations did not change when scores on the control animations were also entered into the regression equation as a control variable, β = 0.24, *t* = 3.30, *p* = 0.001. These findings replicate the results of Study 1, and suggest that participants' general ability to infer human content from the animations is not responsible for the association between their likelihood of recognizing the secure base script and their knowledge of the indicators of a partner who is apt to be there when needed.

#### Organization of script responses

As in Study 1, a regression analysis was performed to predict scores on the organization of the descriptions. Ready recognition of the script across the four animations was a significant predictor (β = 0.60, *t* = 9.01, *p* < 0.001). Another regression analysis was performed to predict scores on the causal connections across the four animations. Ready recognition of the script across the four animations was a significant predictor (β = 0.58, *t* = 8.50, *p* < 0.001). Thus, as in Study 1, participants who recognized the secure base script in the secure base animations provided descriptions of those animations that were better organized. Finally, a regression analysis was performed to predict scores on the number of action units that the participants had used to describe animations across the four animations. Ready recognition of the script across the four animations was a significant predictor (β = 0.22, *t* = 2.64, *p* = 0.01).

Taken together, these results (like results of Study 1) suggest that the stimuli presented in the animations can be conceptualized as a script. Recognizing the underlying script structure seems to help a person to organize an otherwise ambiguous collection of stimuli in the animations into a meaningful story. This organization, in turn, is associated with how a person perceives, interprets, and remembers those stimuli.

#### Associations with ECR

Table 1 presents results of two separate regression analyses predicting the two measures of knowledge (KNOWI accuracy and secure base animation scores). As can be seen, neither attachment-related avoidance nor attachment-related anxiety predicted either measure of knowledge (both predictors were entered simultaneously).

**Table 1:** **Regression analyses predicting the two measures of knowledge**.

**ECR Scale**	**KNOWI Accuracy Scores^a^^,^^b^**		**Secure Base Animation Scores^c^**	
	**β**	***t***	**β**	***t***
Avoidance	−0.06	−0.53	−0.05	−0.37
Anxiety	0.01	0.10	0.14	0.95

a *N = 63*.

b *KNOWI-Accuracy scores, KNOWI-Readiness scores were also entered as a control variable*.

c *N = 51*.

#### Supplementary analyses

In supplementary analyses, we observed that only a subset of the ECR items actually do concern giving or receiving support in times of need (being there). Therefore, the author and a graduate research assistant sorted the ECR items into two categories: those that do concern support (four avoidance items and one anxiety item) and those that do not (The two coders' judgments were identical for all of the items except for one, which resulted in Kappa = 0.87. The disagreement about the one item was resolved by discussion among the coders). The mean of these five items concerning being there was used in the analyses below (Cronbach's α = 0.84).

Two separate regression analyses were conducted to predict the two measures of knowledge (KNOWI accuracy and secure base animation scores) using the following predictors entered simultaneously: (a) the subset of ECR items that concern being there, (b) the avoidance items not concerned with being there, and (c) the anxiety items not concerned with being there. As seen in Table 2, the subset of ECR items that concern being there showed a significant association in the expected direction with both the KNOWI accuracy scores and the secure base animation scores. On the other hand, avoidance and anxiety items not concerned with being there did not predict either measure of knowledge.

**Table 2:** **Regression analyses predicting the two measures of knowledge**.

**ECR Scale**	**KNOWI Accuracy Scores^a^^,^^b^**		**Secure Base Animation Scores^c^**	
	**β**	***t***	**β**	***t***
Avoidance items not concerning being there	0.16	1.34	0.14	0.92
Anxiety items not concerning being there	−0.07	−0.63	−0.00	−0.02
Items concerning being there	−0.36^**^	−3.06	−0.36^*^	−2.36

a *N = 63*.

b *KNOWI-Accuracy scores, KNOWI-Readiness scores were also entered as a control variable*.

c *N = 51*.

* *p < 0.05*.

** *p < 0.01*.

These results suggest that the ECR contains three distinct components: one related to being there, one related to other aspects of attachment-related avoidance, and one related to other aspects of attachment-related anxiety. It should be noted, however, that these were supplementary analyses. The original scales of attachment-related avoidance and attachment-related anxiety did not predict either measure of knowledge. Furthermore, the associations between the being there component of attachment and the two measures of knowledge are modest. Thus, it seems that attachment security is not equivalent to having knowledge about processes related to support. If this assertion is true, an interesting question that future research can address is the following: “Do knowledge and attachment security explain independent aspects of relational outcomes for a person?” In addition, knowledge and attachment security may explain independent portions of the variance in the same outcome variable.

In order to explore the possibility that different aspects of attachment orientations can be broken down into different, theoretically meaningful parts, first a regression analysis was performed predicting the ECR items concerning being there using the following independent variables: (a) desire (motivation) for support, (b) generalized expectations for support, (c) knowledge of indicators of supportiveness, and (d) knowledge of the secure base script. As shown in the first column in Table 3, three factors had significant *unique* contributions: script knowledge, expecting others to be supportive, and motivation for supportive partners, whereas indicator-knowledge was not a significant predictor in this multivariate model. The multiple R was 0.61. These exploratory analyses suggest the possibility that attachment concerns related to support can be broken down into different, theoretically meaningful parts. Table 3 also presents results of similar regression analyses predicting the remaining avoidance and anxiety items that do not concern support. In these regression models no variable was a significant predictor of the remaining anxiety items, and the only significant predictor of the remaining avoidance items was generalized expectations for support. The association between generalized expectations for support with items concerning being there and with the remaining avoidance items is suggested by the theoretical model by Murray et al. (2006), who propose that negative expectations make a person less likely to approach others.

**Table 3:** **Three separate regression analyses predicting (a) the mean of the ECR items concerning being there, (b) avoidance items not concerning being there, and (c) anxiety items not concerning being there**.

	**Being there items**		**Remaining avoidance items**		**Remaining anxiety items**	
	**β**	***t***	**B**	***T***	**β**	***t***
Knowledge of indicators of supportiveness	−0.04	−0.24	0.24	1.38	0.05	0.28
Knowledge of the secure base script	−0.31^*^	−2.23	0.10	0.65	0.22	1.34
Motivation for support	−0.35^*^	−2.38	0.15	0.89	0.19	1.08
Generalized expectations for support	−0.36^**^	−2.75	−0.42^**^	−2.87	−0.26	−1.65

* *p < 0.05*.

** *p < 0.01*.

## General discussion

In both studies, many participants recognized the secure base script in the animations and described the animation using this script. As hypothesized, in Study 1 both measures of participants' ready recognition of the secure base script (the mean of the three clip scores on secure base scriptedness and the common theme score) predicted (a) the proportion of the action units that they recalled, (b) how well organized their descriptions of the animations were, and (c) how many causal connections they used in their descriptions. These findings were replicated in Study 2 (with the exception of recall of action units, which was not assessed in Study 2). In Study 1, both measures of ready recognition of the secure base script (the mean of the three clip scores on secure base scriptedness and the common theme score) were significantly associated with indicator knowledge. This association between the recognition of the secure base script and indicator knowledge was replicated in Study 2, controlling for recognition of control animations.

Attachment-related avoidance and attachment-related anxiety were not associated with ready recognition of the secure base script or with indicator knowledge. However, the subset of attachment items that concern being there showed a significant association in the expected direction with both indicator knowledge and ready recognition of secure base animation scores. In multivariate analyses predicting the attachment items concerning being there, three factors had significant *unique* contributions: script knowledge, expecting others to be supportive, and motivation for supportive partners.

These findings are in line with the social-cognitive model, which suggests that people develop knowledge of interpersonal interaction patterns, which shape processing of information in interpersonal relationships, affecting relationship outcomes (Baldwin, 1992; Fehr, 2005). The present studies are based on the assumption that judgments about new relationship partners are shaped by these knowledge structures (Baldwin, 1992; Fehr, 2005). The present findings indicate that these two types of knowledge (indicator knowledge and script knowledge) are correlated, presumably because they are part of a broader larger knowledge system concerning a close relationship partner's support. There are probably a number of other knowledge structures contained in the broader knowledge system, and the structure, content, and function of those structures could also be explored in future studies using methods similar to the ones used in the present studies. Furthermore, it is possible that emotions also affect judgments about potential partners and this issue should also be examined in future studies. Finally, future studies can examine the effects of these knowledge structures on actual relationship outcomes, such as relationship satisfaction, commitment, and relationship dissolution. One limitation of the present studies is that participants were all undergraduate students. This may limit the generalizability of the findings, and future studies can address this issue by replicating the present results in samples that are older, less educated, and come from diverse socio-economic backgrounds. Another limitation is the use of fixed order for the animations (as opposed to randomized order across participants).

Nevertheless, results suggest that many people recognize the secure base script from minimal cues. Thus, the knowledge of the script does not seem to be limited to the context of existing close relationships: People can recognize the abstract and generalized structure of the script that they can apply to any close relationship. The present results also demonstrate how script knowledge shapes memory and processing of relevant information. Surprisingly, however, not everyone readily recognizes and uses the secure base script (or knows the indicators of a partner who is apt to be there when needed). The ready recognition of the script may have important effects in people's everyday lives. A person who readily recognizes the script should be in a better position to recognize when support is appropriate—both as a seeker and as a provider. The person should also recognize when a partner is apt to provide quality support. This recognition, in turn, may allow the person to benefit more from support exchanges, which are known to contribute to psychological and physical well-being (Barrera, 2000; Uchino, 2006). In addition, recognizing the script with ease may enable a person to make more accurate global judgments about a partner and contribute to the development of trust in a relationship.

It is possible that a person needs to use *both* knowledge structures simultaneously in order to make judgments about a partner. Indicator-knowledge concerns acts (behaviors) of a person who is apt to be there when needed, whereas script-recognition specifies the context (situation) in which those acts produce the desired outcome. For example, “supportive” acts that are delivered in an inappropriate context do not constitute genuine support—e.g., offering comfort or consolation to a very private person at a public gathering. Similarly, the wrong act in a context that calls for secure-base support might not be genuinely supportive—e.g., providing instrumental help when secure-base support is wanted.

The animation method used to assess recognition of the secure base script has advantages as an assessment tool. The responses are easy for participants to produce, and relatively easy for a researcher to code. In future studies, recognition of other interpersonal scripts (see Baldwin, 1992) for examples such as asking someone for a date, disclosing embarrassing information about oneself, trying to cheer a depressed person) could also be tested using a similar method involving animations.

As noted previously, the knowledge structures examined in this paper are theoretically distinct from attachment security. Attachment security is a broad concept and includes components such as generalized positive expectations concerning relationships, a relative lack of defensiveness, and appropriate emotion regulation strategies (due to low attachment-related avoidance and low attachment-related anxiety). Therefore, indicator knowledge and secure base script-recognition open up the possibility for examining the *independent* effects of these knowledge structures on the one hand, and attachment security on the other, on people's actual relationships. For example, one study (Turan et al., 2011) examined the accuracy of surrogate decision makers in predicting an older relative's end-of-life health care wishes and found that both indicator-knowledge and script-knowledge of the surrogates explain additional variance in their accuracy over and above the variance explained by their attachment security. Similarly, another study (Turan and Vicary, 2010) found independent effects of indicator knowledge and attachment security on people's ability to identify relationship-enhancing behaviors. Given the well-known strong effects of social relationships on mental and physical health (see for example a review by House et al., 1988), it is important to understand factors that affect close relationships and develop interventions aimed at improving them. Targeting knowledge structures related to support may be one of the many different components of comprehensive multi-modal interventions aimed at improving a person's social relationships.

## Author contributions

The author confirms being the sole contributor of this work and approved it for publication.

### Conflict of interest statement

The author declares that the research was conducted in the absence of any commercial or financial relationships that could be construed as a potential conflict of interest.
